# Book Notices

**Published:** 1884-09

**Authors:** 


### BOOK NOTICES.

THE THERAPEUTICS OF INTERMITTENT FEVER. By H. C. Allen, M. D., University of Michigan. Octavo; pages 340; price, $2.75. Philadelphia: Hahnemann Publishing House, F. E. Boericke, 1884.

The first edition of Dr. Allen's work on intermittent fevers, issued some years ago, speedily won a great reputation among homœopathists. Of this, the second edition, it is therefore merely necessary to say that it is very much improved and enlarged. The former edition gave only the therapeutics of intermittent fever in its various stages; to this the new work adds the characteristics of each remedy, together with a fine repertory.

The book is excellently well gotten up, and is another one of those homœopathic works which every physician should purchase. A more extended notice of this valuable book is unnecessary, as we feel sure all of our readers will speedily examine it for themselves.

CHOLERA: ITS PREVENTION AND TREATMENT. By D. N. Rây, M. D., L. S. A. (London). Pages 126; price, $1.00. New York: A. L. Chatterton Publishing Company, 1884.

In this essay Dr. Rây has given a most thorough and timely monograph on cholera. He gives a full history of its ravages, a complete *resume* of its ætiology, with the numerous theories on this subject, its causes, symptoms, its sequelæ and treatment. In writing of the treatment mention is made of the various futile prescriptions of the old school. The comparative results of the two systems gives the *average* of all allopathic methods at seventy per cent., while the highest mortality under homœopathic treatment is about fifteen per cent.

The therapeutics of the homœopathic remedies could have been stated much more thoroughly. The attempt to fix the dose for each medicine was unnecessary and useless.

In this connection it may be interesting to quote from the report of the American Consul on the results of "scientific medicine" in the treatment of cholera now raging in the South of France. We hope later on to give reports of the homœopathic treatment from some of our colleagues resident in the infected district:

"From the 30th of June until the 10th of July, four hundred and thirty cases of cholera were officially recorded at Marseilles. Of these two hundred and ninety-four died, and the remaining one hundred and thirty-six were, at the date of the report from which these figures are quoted, in various stages of convalescence. It therefore appears that, notwithstanding all progress in medical science and the very perfect arrangements for collecting and treating the victims of the scourge, more than two-thirds of those attacked have died, even during the first fortnight of the epidemic, when all sanitary conditions were most favorable. These two facts, the almost immediate transmission of the disease from Toulon to Marseilles, and the enormous death rate of seventy per cent. in the earliest stage of the epidemic, seem to prove that sanitary science and medical skill have made but little substantial progress in dealing with Asiatic cholera."

THE PROCEEDINGS OF THE EIGHTH ANNUAL SESSION OF THE MISSOURI INSTITUTE OF HOMŒOPATHY.

THE TRANSACTIONS OF THE FIFTEENTH ANNUAL SESSION OF THE HOMŒOPATHIC MEDICAL SOCIETY OF MICHIGAN.

Both of these volumes are interesting and make a creditable showing for their respective societies.

"DRUGS AND MEDICINES OF NORTH AMERICA." A quarterly devoted to the historical and scientific discussion of the botany, pharmacy, chemistry, and therapeutics of the medicinal plants of North America. By J. U. & C. G. Lloyd. Cincinnati: 180 Elm Street. 1884.

The above quoted title gives one a clear idea of the purposes of this quarterly. The descriptive text is illustrated by wood-cuts. Subscription price is only one dollar a year, which brings a most valuable work within the reach of all. Though the homœopathic value of these plants is not well defined, being given chiefly by Dr. E. M. Hale, nevertheless the work is worth much more than the subscription price.

---

A GOOD INVESTMENT:—Any of our readers who desire to make a paying investment should send five dollars to 113 Fulton Street, New York city, and secure a copy of *The Sanitarian*. It is certainly a most valuable monthly.

